# Primary signet-ring cell carcinoma of the urinary bladder successfully managed with cisplatin and gemcitabine: a case report

**DOI:** 10.1186/1752-1947-7-37

**Published:** 2013-02-06

**Authors:** Jalal Eddine El Ammari, Mustapha Ahsaini, Omar Riyach, Mohammed Jamal El Fassi, My Hassan Farih, Nawal Hammas, Hind Elfatmi, Afaf Amarti

**Affiliations:** 1 Department of Urology, University Hospital Center Hassan II, Route Sidi Hrazem, Fez, 30000, Morocco; 2 Department of Pathology, Hospital University Center Hassan II, Route Sidi Hrazem, Fez, 30000, Morocco

**Keywords:** Primary signet-ring cell carcinoma, Adenocarcinoma, Urinary bladder, Chemotherapy

## Abstract

**Introduction:**

Primary signet-ring cell carcinoma of the urinary bladder is a rare variant of mucus-producing adenocarcinoma constituting approximately 0.5% to 2.0% of all primary carcinomas of the bladder. This tumor initially presents as a high-grade, high-stage lesion and diffusely invades the bladder wall without forming intraluminal growth. The patients have no specific symptoms, which leads to delayed diagnosis and poor prognosis.

**Case presentation:**

We report the case of a 51-year-old Moroccan Berber man consulting for gross hematuria. Ultrasonography and a computed tomography scan found a bladder tumor diffusely invading the bladder wall. A histopathological examination of the tumor chips from a transurethral resection of the bladder revealed signet-ring cell adenocarcinoma. The gastrointestinal tract exploration did not reveal any other tumor localization. A radical cystectomy and adjuvant cisplatin and gemcitabine chemotherapy were therefore performed resulting in 18 months of survival without metastasis and a good quality of life within that time.

**Conclusion:**

The rarity and the successful management with carboplatin and gemcitabine as adjuvant chemotherapy of this entity, which is rarely reported in the literature, are two remarkable characteristics described in this case report.

## Introduction

Primary signet-ring cell carcinoma (PSRCC) is a rare neoplasm of the urinary bladder. Less than 100 cases were described after Saphir reported the first two cases in 1955 [1]. We present one case of primary signet-ring cell carcinoma of the urinary bladder with a brief review of the current literature.

## Case presentation

**Figure 1:**
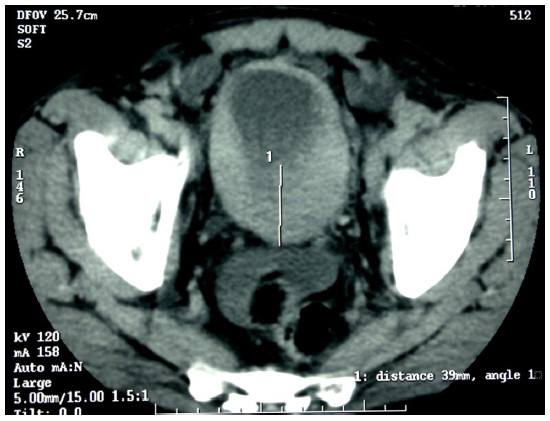
Pelvic computed tomography showing an invasive tumor of the urinary bladder.

**Figure 2:**
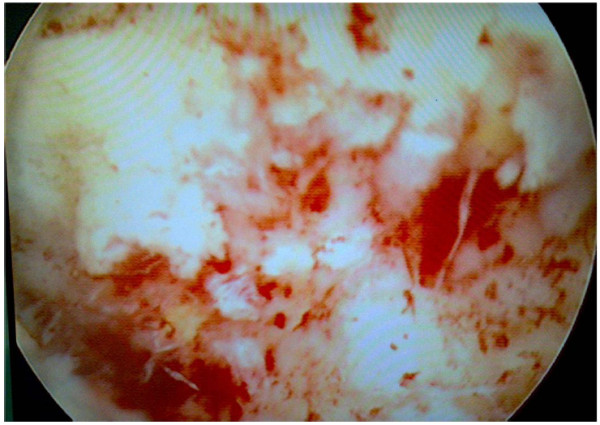
Cystoscopic appearance showing multiple grape-like lesions in the posterior wall of the urinary bladder.

**Figure 3:**
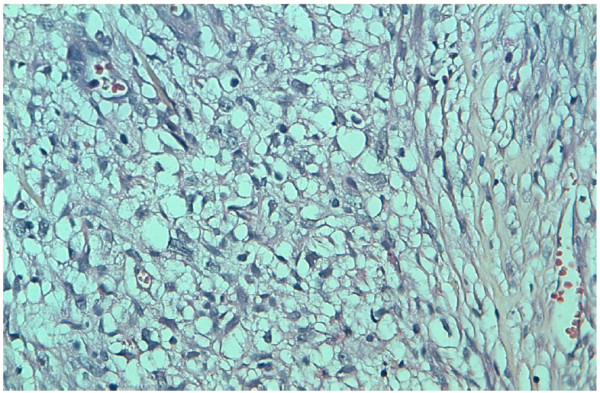
Histopathology of the bladder lesion showing multiple signet-ring cells in the lamina propria with overlying transitional cell epithelium.

A 51-year-old Moroccan Berber man was referred to our hospital with a history of three months of intermittent painless total gross hematuria, frequency and urgency. The medical and familial histories were unremarkable. Ultrasonography revealed a grade II left hydronephrosis and circumferential thickening of the urinary bladder wall. A computed tomography (CT) scan showed a diffuse neoplasm of the urinary bladder with no lymph node enlargement or distant metastases (Figure 1). Cystoscopic examinations revealed nonpapillary sessile tumors occupying almost the whole of the bladder wall (Figure 2), and the left side of the trigone obstructing the left ureteral meatus. Transurethral resection of the lesion was realized incompletely because of the diffuse character of the tumor. Histopathological specimen examination found a poorly differentiated mucin-secreting adenocarcinoma of the signet-ring cell type (Figure 3).

We performed a complete gastrointestinal endoscopic evaluation and analysis of tumor markers to exclude an extravesical primary tumor site, but no other primary site was found. The tumor was therefore treated as a primary signet-ring cell carcinoma of the urinary bladder.

The patient underwent radical cystectomy with an ileal conduit and bilateral pelvic lymphadenectomy. Histopathological examination of the surgical specimen reported adenocarcinoma composed of signet-ring cells with an abundant mucin pool that was invading the perivesical adipose tissue and one lymph node (<2cm) staging the tumor at pT3bN1M0. Adjuvant chemotherapy was then performed with four cycles of cisplatin, a 100mg/m2 on day 1 and gemcitabine 1000mg/m2 on days 1 and 8 combination, every 21 days. The patient was followed up every six months, and a thoracoabdominal CT scan was done every six months. He was free from metastasis, the quality of life was good without symptoms of pain or renal dysfunction, and he achieved weight recovery soon after finishing his chemotherapy. Eighteen months postoperatively, the patient presented with pelvic and back pain. Multiple metastases to spine, lung and pelvis were noted on a thoracoabdominal CT scan. The patient refused to be enrolled in any additional protocol and died four months later due to respiratory distress.

## Discussion

Primary signet-ring cell carcinoma of the urinary bladder is a relatively rare subtype of adenocarcinoma and comprises only 0.24% to 2% of all primary epithelial urinary bladder tumors [2,3]. Less than 100 cases have been reported in the literature since the first two cases reported by Saphir in 1955 [1]. The histopathogenesis of primary mucin-producing adenocarcinomas, including signet-ring cell carcinomas, remains unclear, because the normal bladder contains neither columnar nor mucus-secreting glandular epithelium. Many investigators proposed that the metaplastic potential of the urothelium has two distinct patterns. Progressive invagination of hyperplastic epithelial buds into the lamina propria (Von Brunn’s nest) leads to the formation of cystitis cystica. Subsequent metaplasia of the urothelial lining within these cysts to columnar mucin-producing cells results in the production of cystitis glandularis, which is a premalignant lesion. Alternatively, cuboidal or columnar metaplasia of the surface epithelium can occur without downward invagination, with chronic vesical irritation and infection being the predisposing factors of these changes. Most of these tumors are mucin-secreting but the passage of mucus during micturition is uncommon. Two-thirds of the tumors were mucin-secreting, in most of which the site of the deposition was extracellular (interstitial). Less commonly, mucin is secreted within the lumen of the acini and infrequently, excessive intracellular mucin displaces the nucleus to a peripheral crescent, giving the cells a signet-ring appearance [4].

Generally, this neoplasm occurs in middle age and is usually diagnosed at an advanced stage, usually demonstrating a subsequently poor prognosis [5]. The usual clinical presentation does not differ significantly from that of other bladder malignancies [6]. The common presenting symptoms were irritative voiding symptoms and hematuria. Urinary retention and flank pain due to ureteral obstruction were less common. Signet-ring cell carcinomas of the bladder were most often infiltrative and diffusely involving the majority of the bladder. The lesion is described in cystoscopy as pedunculated, polypoid, sessile, and ulceroinfiltrative [4]. Bladder adenocarcinoma may arise in any region of the bladder but it is usually found in the bladder dome [6]. It may be very difficult to rule out because it has the same histological and immunohistochemical features as urachal carcinoma. Signet-ring cells can also be found in adenocarcinomas of urachal origin. Several criteria for classifying a tumor as urachal in origin have been suggested [2]: 1) tumor in the bladder (dome), 2) a sharp demarcation between the tumor and the surface epithelium, and 3) exclusion of primary adenocarcinoma located elsewhere that spread secondarily to the bladder. The present case showed no sharp demarcations between the tumor and the surface epithelium. Thus, we could exclude an urachal tumor in origin.

It is essential to distinguish this carcinoma from metastases, as different therapeutic strategies are often necessary. Primary signet-ring cell carcinoma of the urinary bladder has the same histology as that of the gastrointestinal tract, breast, lung, gallbladder, and prostate; therefore, further evaluations for other primary sites are mandatory to exclude metastasis [4,6]. In our case, the gastrointestinal evaluation included esophagogastroduodenoscopy and colonoscopy, but we found no other tumor lesions. Although there is no established serum marker of primary signet-ring cell carcinoma of the urinary bladder, elevated carcinoembryonic antigen (CEA) has often been reported. Yamamoto et al. [4] reported that the serum level of CEA is normalized postoperatively and gradually increases as the disease progresses. They have suggested, therefore, that CEA might be used for determining malignant potential and for monitoring signet-ring cell carcinoma. In our case, CEA elevation was not noted.

The prognosis is usually poor as it is usually diagnosed at an advanced stage [1-7]. Treatment modalities for signet-ring cell carcinomas include surgery, radiotherapy, and chemotherapy. Surgical options range from transurethral resection to radical cystectomy with urinary diversion. According to the review by Erdogru et al. [7] only total cystectomy might offer some hope for patients. Several effective treatments including intra-arterial chemotherapy with cisplatin and methotrexate [8] and radiation therapy, or only radiotherapy after cystectomy were reported in the literature. Unfortunately, no standard chemotherapy exists for PSRCCs of the bladder because of their rarity. We report this case of PSRCC treated with total cystectomy followed by systemic chemotherapy with cisplatin and gemcitabine, a standard combination for transitional carcinoma of the urinary bladder, with a successful outcome due to the time of survival without metastasis, which was 18 months, and 22 months before he died.

## Conclusion

Primary signet-ring cell carcinoma of the urinary bladder is a rare histological variant of mucus-producing adenocarcinoma, which is generally high-grade, high-stage and has a uniformly poor prognosis. Recently, successful treatments with chemotherapy alone have been reported. However, the appropriate regimen and the method of injection have not yet been established. This case is unique in using other kinds of chemotherapy (cisplatin and gemcitabine) resulting in 18 months of survival without metastasis.

## Consent

Written informed consent was obtained from the patient’s next-of-kin for publication of this case report. A copy of the written consent is available for review by the Editor-in-Chief of this journal.

## Competing interests

The authors declare that they have no competing interests.

## Authors’ contributions

JA, MA and OR were the principal authors and major contributors in writing the manuscript to JA, MA were the principal authors and major contributors in writing the manuscript. NH, and HE analyzed and interpreted the patient data and reviewed the literature to OR, NH, and HE analyzed and interpreted the patient data and reviewed the literature. All authors read and approved the final manuscript.
